# Supplementary material for: The E3 ubiquitin ligase mechanism specifying target-directed microRNA degradation
Source: bioRxiv. 2026 Jan 5:2026.01.05.697729. Preprint. [Version 1] doi: 10.64898/2026.01.05.697729 (PMC12803205; doi:10.64898/2026.01.05.697729)
Supplement: Supplement 3 [file media-3.pdf]

Cryo-EM data collection, refinement and validation statistics

	Map A ZSWIM8-CUL3 complex bound to AGO2-miR-7-CYRANO,	Map B Locally refined interactions of ZSWIM8-CUL3 complex bound to AGO2-miR-7-CYRANO	Map C focused map of ZSWIM8-CUL3 complex bound to AGO2-miR-7-CYRANO	Map D Locally refined map of ZSWIM8-CUL3 complex bound to AGO2-miR-7-CYRANO	Map E Composite map of ZSWIM8-CUL3 complex bound to AGO2-miR-7-CYRANO
**Data collection and processing**					
Magnification	105,000	105,000	105,000	105,000	105,000
Voltage (kV)	300	300	300	300	300
Electron exposure (e–/Å^2)	58	58	58	58	58
Defocus range (μm)	-0.5 – -2.0	-0.5 – -2.0	-0.5 – -2.0	-0.5 – -2.0	-0.5 – -2.0
Pixel size (Å)	0.8512	0.8512	0.8512	0.8512	0.8512
Symmetry imposed	C1	C1	C1	C1	C1
Initial particle images (no.)	5,812,715	5,812,715	5,812,715	5,812,715	5,812,715
Final particle images (no.)	234,181	234,181	234,181	234,181	234,181
Map resolution (Å)	3.1	3.1	3.2	3.2	3.1
FSC threshold					
Map resolution range (Å)	2.8-3.3	2.7-3.2	2.8-3.3	2.8-3.4	2.8-3.3
**Refinement**					
Initial model used (PDB code)					Alphafold3, AGO2 (6NIT), CUL3 (5NLB), ELOB/C (1LM8)
Model resolution (Å)					3.1
FSC threshold					
Model resolution range (Å)					
Model composition					
Non-hydrogen atoms					28,597
Protein residues					3,609
Nucleotides					50
Ligands					2 Zn
B factors (Å^2)					
Protein					87.42
Nucleotides					103.12

Ligand	97.48
R.m.s. deviations	
Bond lengths (Å)	0.007
Bond angles (°)	0.603
Validation	
MolProbity score	2.02
Clashscore	5.75
Poor rotamers (%)	2.10
Ramachandran plot	
Favored (%)	92.77
Allowed (%)	7.14
Disallowed (%)	0.08
