# Supplementary material for: The E3 ubiquitin ligase mechanism specifying target-directed microRNA degradation
Source: bioRxiv. 2026 Jan 5:2026.01.05.697729. Preprint. [Version 1] doi: 10.64898/2026.01.05.697729 (PMC12803205; doi:10.64898/2026.01.05.697729)
Supplement: Supplement 14 [file NIHPP2026.01.05.697729v1-supplement-14.pdf]

bioRxiv preprint doi: https://doi.org/10.64898/2026.01.05.697729; this version posted January 5, 2026. The copyright holder for this preprint (which was not certified by peer review) is the author/funder, who has granted bioRxiv a license to display the preprint in perpetuity. It is made available under aCC-BY 4.0 International license.

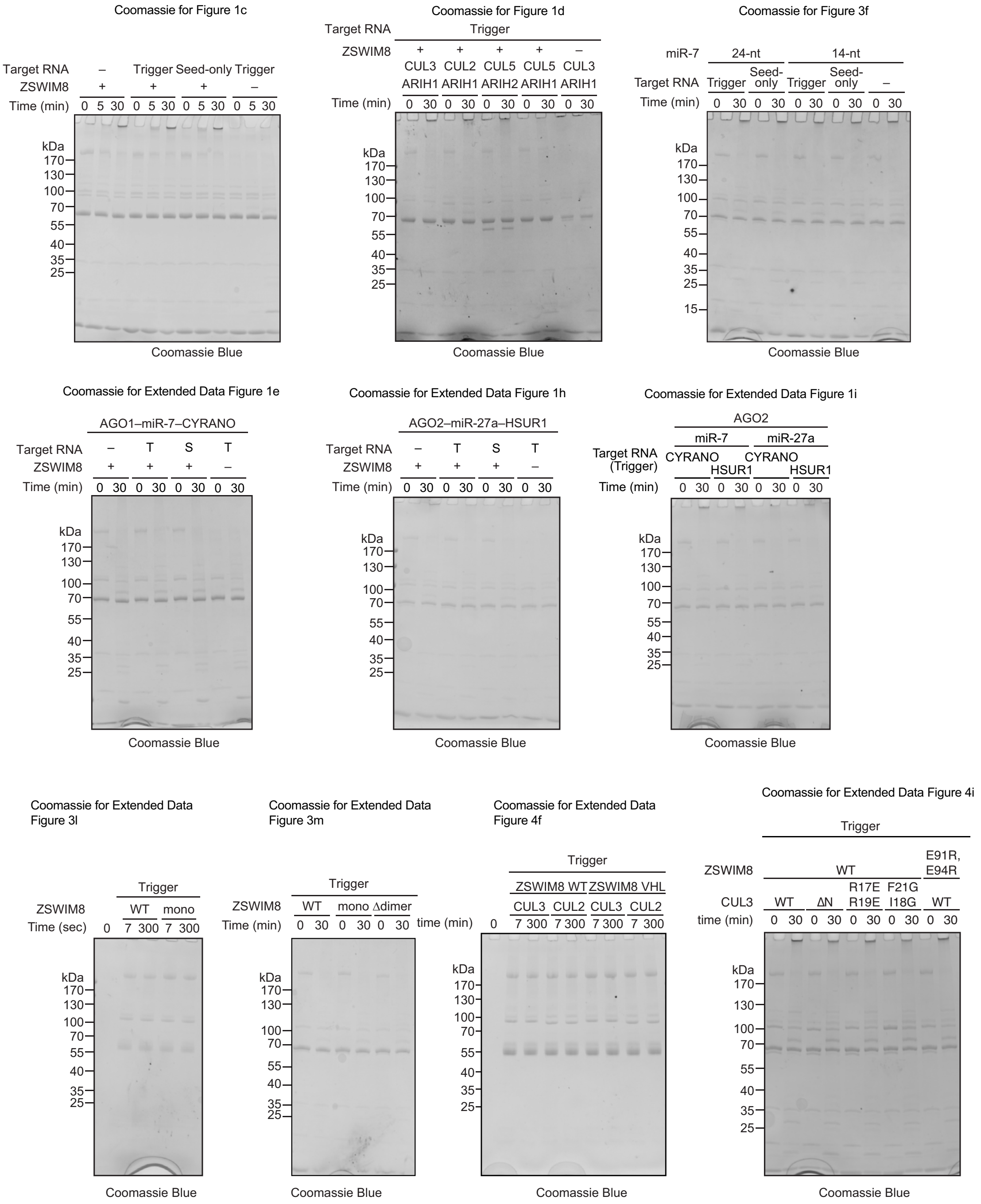

Supplementary Figure 1. Gels stained with Coomassie Blue for ubiquitylation assays shown in Figure 1c, 1d, and 3f, and Extended Data Figures 1e, 1h, 1i, 3l, 3m, 4f, and 4i.

bioRxiv preprint doi: https://doi.org/10.64898/2026.01.05.697729; this version posted January 5, 2026. The copyright holder for this preprint (which was not certified by peer review) is the author/funder, who has granted bioRxiv a license to display the preprint in perpetuity. It is made available under aCC-BY 4.0 International license.

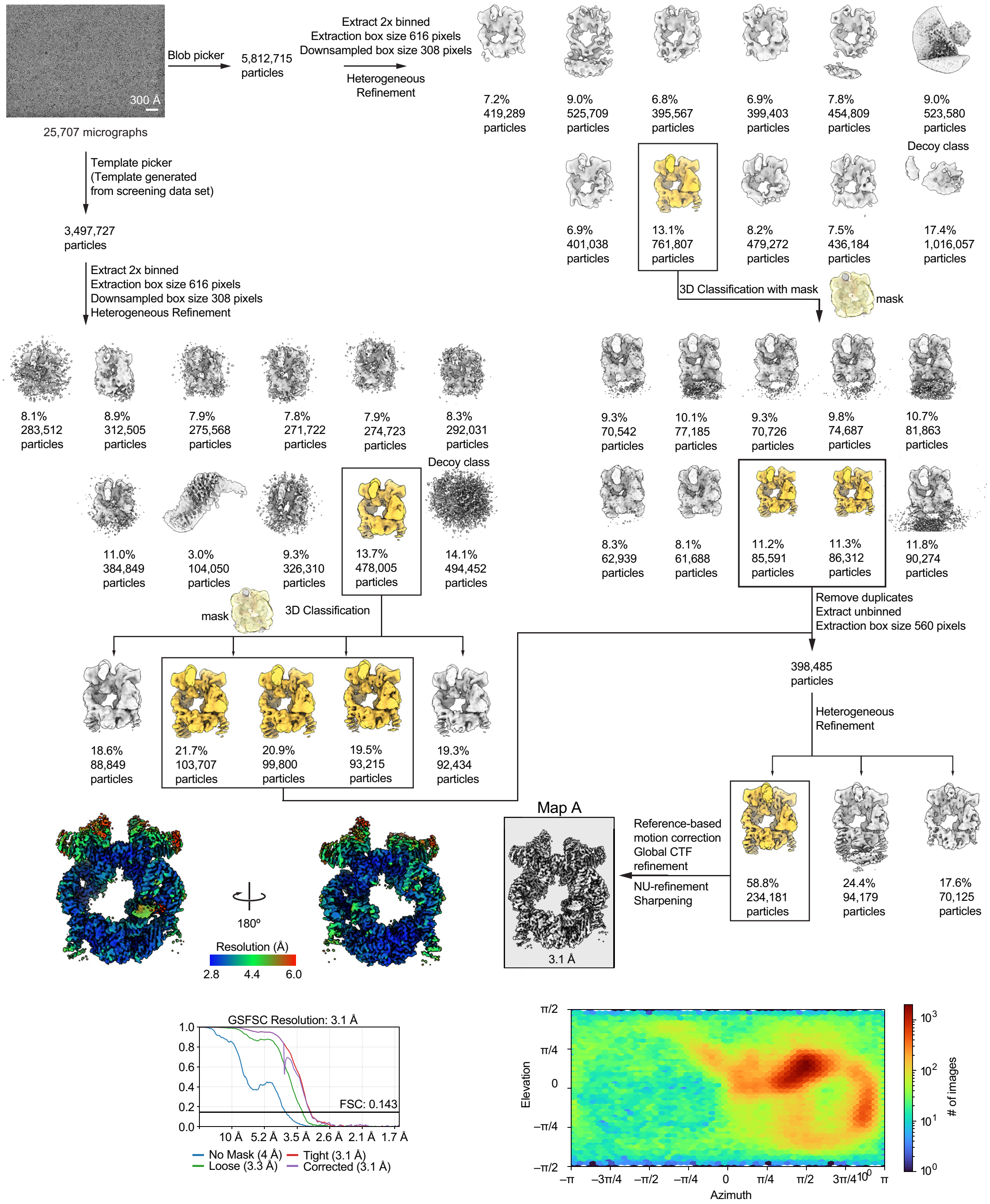

Supplementary Figure 2. Processing scheme for AGO2–miR-7–CYRANO and ZSWIM8–CUL3 complex. Representative micrograph is shown. Classes selected from classification step are shown in yellow. Masks for masked classification are shown in transparent yellow. Heterogeneous refinements contained one decoy class. Local resolution of Map A is shown. Bottom left, Gold-standard Fourier shell correlation (GSFSC) is shown at a cut-off of 0.143 providing a resolution of 3.1 Å. Bottom right, orientation distribution plot of particles used to generate Map A.

bioRxiv preprint doi: https://doi.org/10.64898/2026.01.05.697729; this version posted January 5, 2026. The copyright holder for this preprint (which was not certified by peer review) is the author/funder, who has granted bioRxiv a license to display the preprint in perpetuity. It is made available under aCC-BY 4.0 International license.

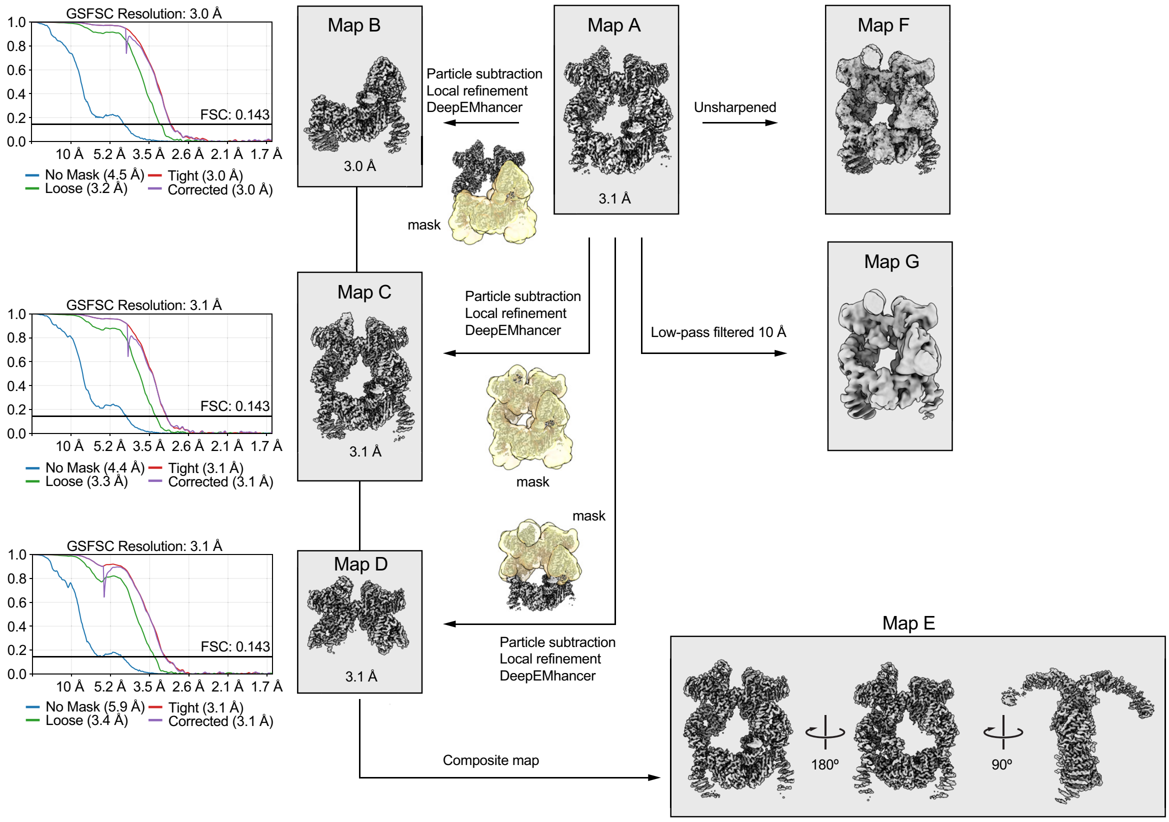

Supplementary Figure 3. Processing scheme to generate maps derived from consensus map A. Focused refinements to generate maps B, C, and D are shown. Masks used for focused refinement are shown in yellow. Gold standard Fourier shell correlation (GSFSC) is shown at a cut-off of 0.143. Composite map E was generated from maps B, C, and D. Unsharpened map F and low-pass filtered map G were derived from consensus map A.

bioRxiv preprint doi: https://doi.org/10.64898/2026.01.05.697729; this version posted January 5, 2026. The copyright holder for this preprint (which was not certified by peer review) is the author/funder, who has granted bioRxiv a license to display the preprint in perpetuity. It is made available under aCC-BY 4.0 International license.

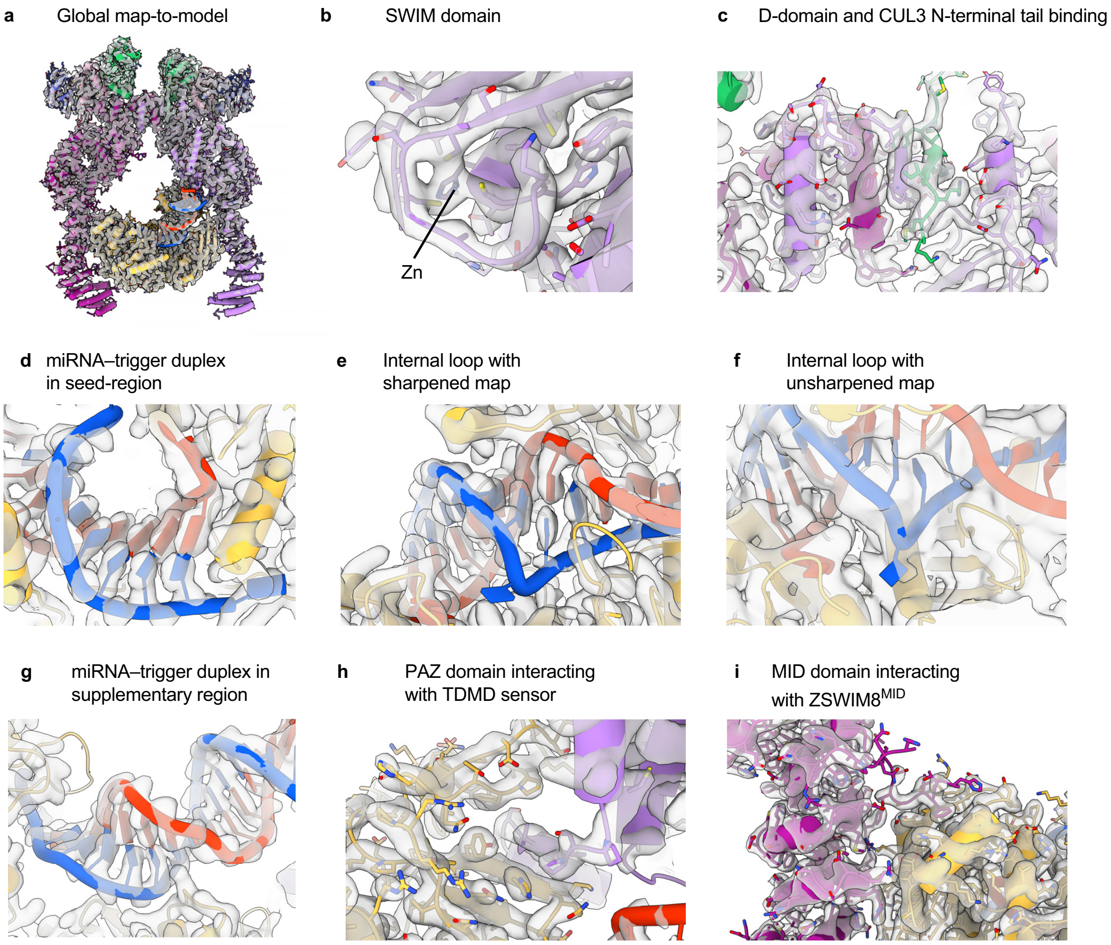

Supplementary Figure 4. Map-to-model fit of composite Map E to the atomic model. For each panel, the model is colored as in Figure 2. **a**, Global map-to-model fit. **b**, Fit of the SWIM domain. The zinc ion is highlighted for clarity. **c**, Fit of the dimerization domain (D-domain) and the associated CUL3 N-terminal tail. **d–g**, Fit of the RNA model to the density in several regions: **d**, seed region; **e**, internal loop with sharpened Map E; **f**, internal loop with unsharpened Map F; **g**, distal region. **h**, Fit of the TDMD sensor of the ZSWIM8^{NPAZ} domain binding to the AGO2 PAZ domain. **i**, Fit of the ZSWIM8^{MID} domain interaction with the AGO2 MID domain.

bioRxiv preprint doi: https://doi.org/10.64898/2026.01.05.697729; this version posted January 5, 2026. The copyright holder for this preprint (which was not certified by peer review) is the author/funder, who has granted bioRxiv a license to display the preprint in perpetuity. It is made available under aCC-BY 4.0 International license.

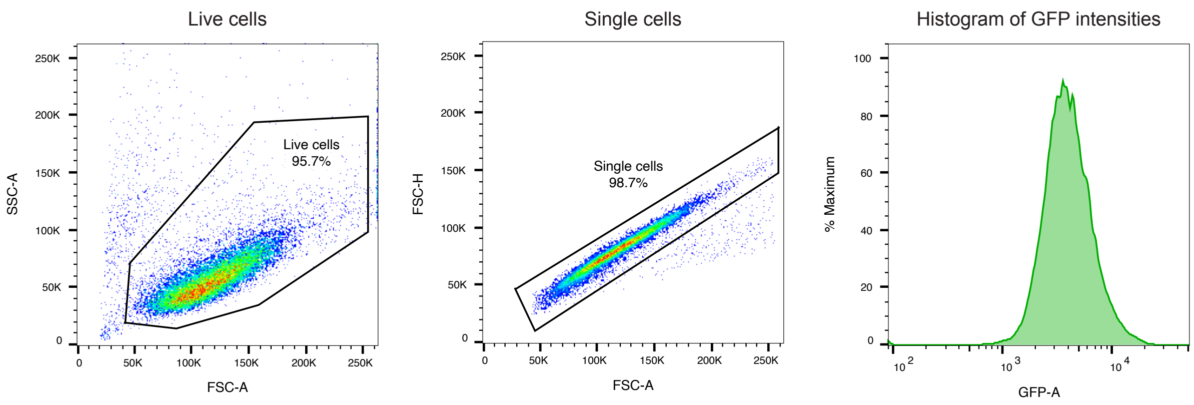

Supplementary Figure 5. Gating strategy for the ZSWIM8 intracellular rescue assay. Shown are representative plots from cells expressing wild-type ZSWIM8. Cells were gated to obtain live, single cells (left and middle, respectively). The GFP fluorescence was recorded for this subpopulation of cells (right). Approximately 20,000 live, single cells were analyzed for each sample.

bioRxiv preprint doi: https://doi.org/10.64898/2026.01.05.697729; this version posted January 5, 2026. The copyright holder for this preprint (which was not certified by peer review) is the author/funder, who has granted bioRxiv a license to display the preprint in perpetuity. It is made available under aCC-BY 4.0 International license.

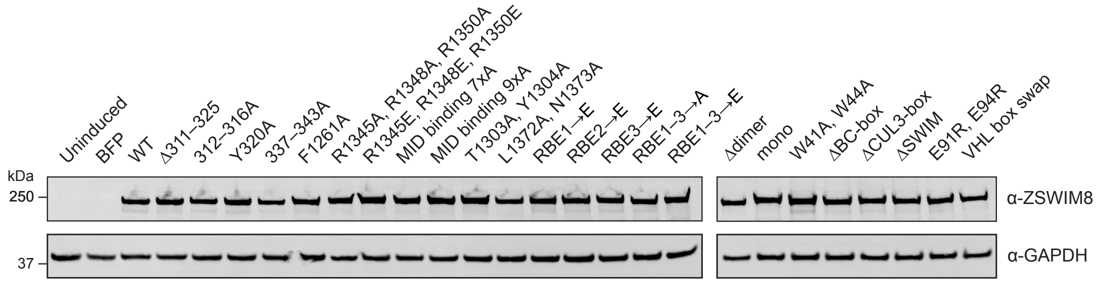

Supplementary Figure 6. Levels of ZSWIM8 protein variants used in the intracellular TDMD reporter assays shown in Figures 3c and 4c, and Extended Data Figures 3e–g, 3n, 4b, and 4j. Shown are representative western blots detecting expression of each ZSWIM8 variant as well as of GAPDH, which served as a loading control. All ZSWIM8 variants were expressed at levels comparable to that of WT ZSWIM8.

bioRxiv preprint doi: https://doi.org/10.64898/2026.01.05.697729; this version posted January 5, 2026. The copyright holder for this preprint (which was not certified by peer review) is the author/funder, who has granted bioRxiv a license to display the preprint in perpetuity. It is made available under aCC-BY 4.0 International license.

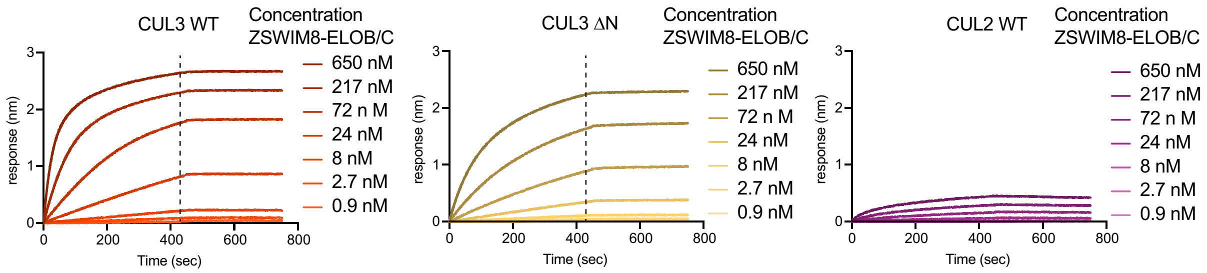

Supplementary Figure 7. Biolayer interferometry measurements to determine binding affinity of ZSWIM8 for CUL3 WT, CUL3 ΔN (lacking residues 1–24), and CUL2 WT N-terminal domains (Extended Data Figure 4h). Sensorgrams were normalized to the start of the association step. The dashed line indicates the time point at which the maximum response was measured and used for the determination of binding affinity. The dissociation step starting at 430 seconds did not induce any dissociation of ZSWIM8.

bioRxiv preprint doi: https://doi.org/10.64898/2026.01.05.697729; this version posted January 5, 2026. The copyright holder for this preprint (which was not certified by peer review) is the author/funder, who has granted bioRxiv a license to display the preprint in perpetuity. It is made available under aCC-BY 4.0 International license.

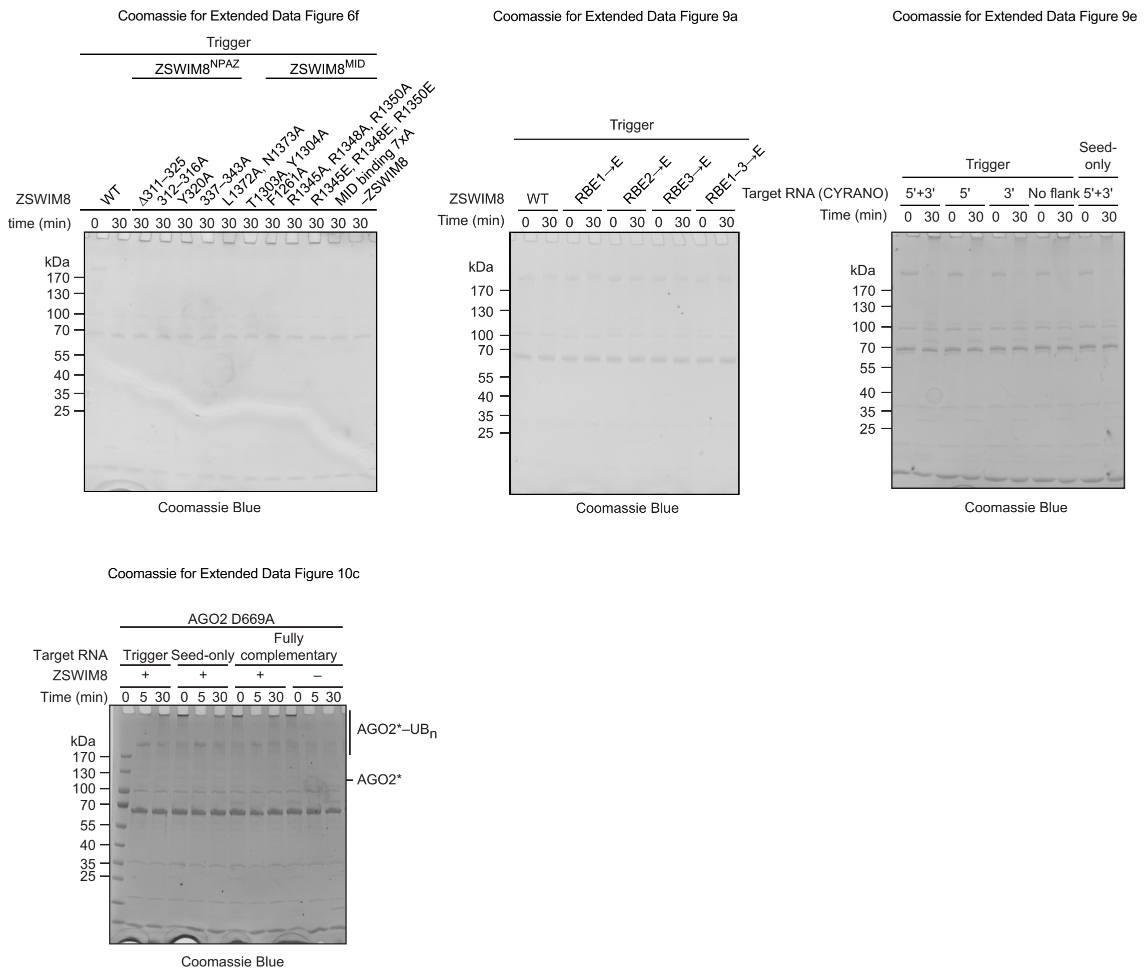

Supplementary Figure 8. Gels stained with Coomassie Blue for ubiquitylation assays shown in Extended Data Figures 6f, 9a, 9e, and 10c.

bioRxiv preprint doi: https://doi.org/10.64898/2026.01.05.697729; this version posted January 5, 2026. The copyright holder for this preprint (which was not certified by peer review) is the author/funder, who has granted bioRxiv a license to display the preprint in perpetuity. It is made available under aCC-BY 4.0 International license.

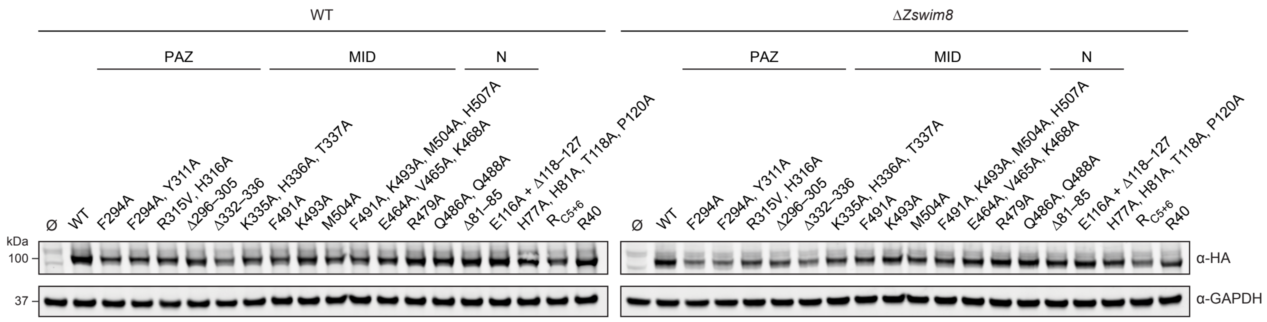

Supplementary Figure 9. Levels of AGO2 protein variants used in the intracellular AGO2 co-IP assay shown in Extended Data Figure 7b and c. Shown are representative western blots detecting expression of each HA-tagged AGO2 variant as well as of GAPDH, which served as a loading control. All AGO2 variants were expressed at levels comparable to that of WT AGO2.

bioRxiv preprint doi: https://doi.org/10.64898/2026.01.05.697729; this version posted January 5, 2026. The copyright holder for this preprint (which was not certified by peer review) is the author/funder, who has granted bioRxiv a license to display the preprint in perpetuity. It is made available under aCC-BY 4.0 International license.

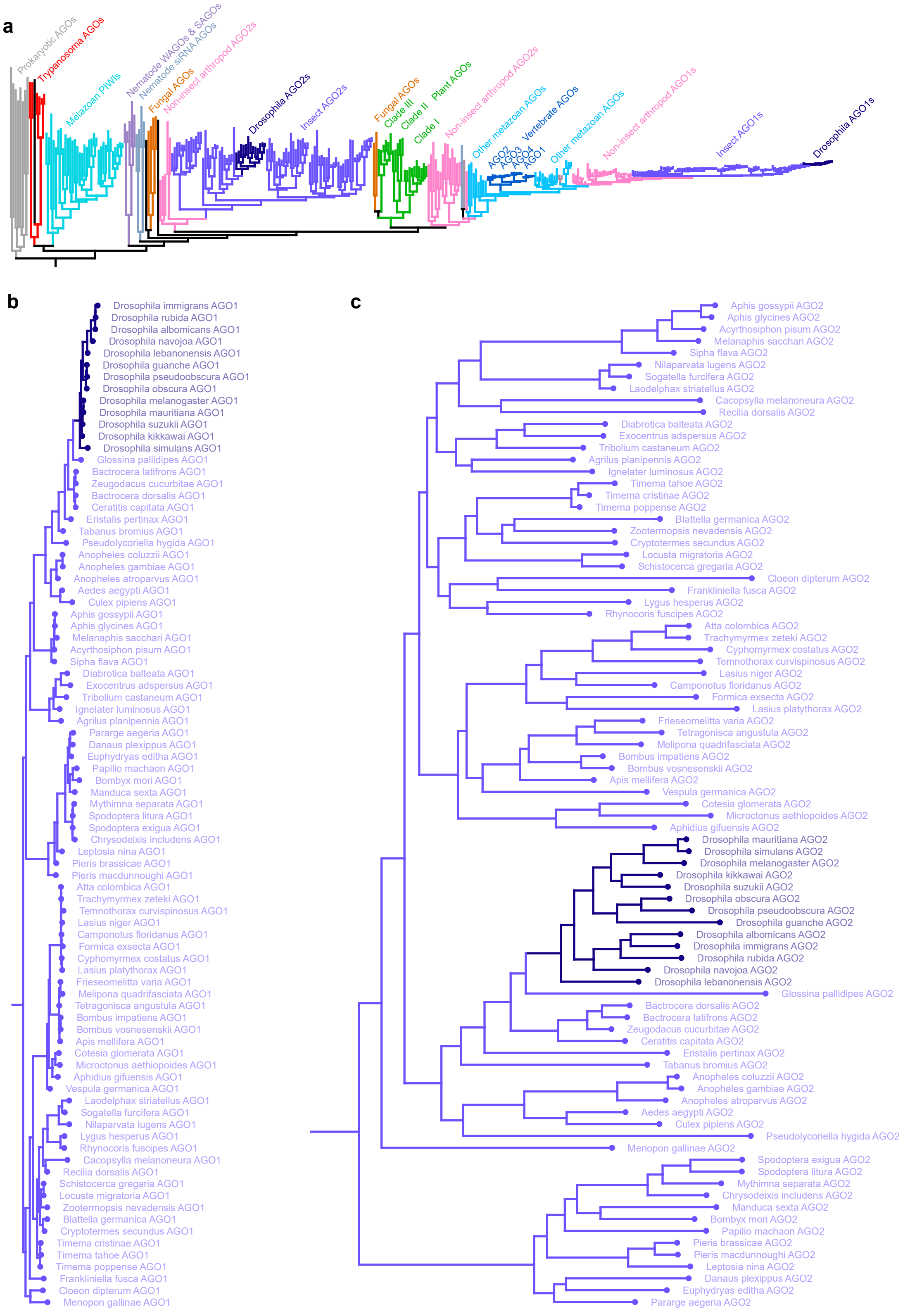

Supplementary Figure 10. Phylogenetic comparison of insect AGO homologs with different TDMD competencies. **a**, Phylogenetic tree of 347 homologs of AGO-family proteins, including some prokaryotic AGOs, Trypanosoma AGOs, nematode-specific AGOs, metazoan PIWIs, and AGOs from plants, fungi, metazoans, and other eukaryotes. Among these homologs are 85 matched pairs of AGO1 and AGO2 homologs in insects, including those from 13 Drosophila species. Phylogeny was calculated using FastTree 2.2 (ref. 86) based on a multiple-sequence alignment of protein sequences obtained from UniProt[87], aligned using the MUSCLE algorithm[88] with the SnapGene software. Homolog groups of interest are labelled and highlighted in distinct colors. Branch lengths are scaled to the rate of amino-acid changes between homologs. **b**, Phylogenetic tree of 85 insect AGO1 proteins, subsetted and magnified from **a**. Colors are as in **a**. Drosophila homologs are highlighted in dark purple. **c**, Phylogenetic tree of 85 insect AGO2 proteins, otherwise as in **b**, from the same 85 insect species and at the same branch-length scale. Branch lengths for insect AGO2 homologs, which are not thought to be subject to TDMD (based on studies of the fly protein)[89], are longer than those for insect AGO1 homologs, which are subject to TDMD[17]. The shorter branch lengths of AGO1 indicates that its sequence is much more evolutionarily constrained.

bioRxiv preprint doi: https://doi.org/10.64898/2026.01.05.697729; this version posted January 5, 2026. The copyright holder for this preprint (which was not certified by peer review) is the author/funder, who has granted bioRxiv a license to display the preprint in perpetuity. It is made available under aCC-BY 4.0 International license.

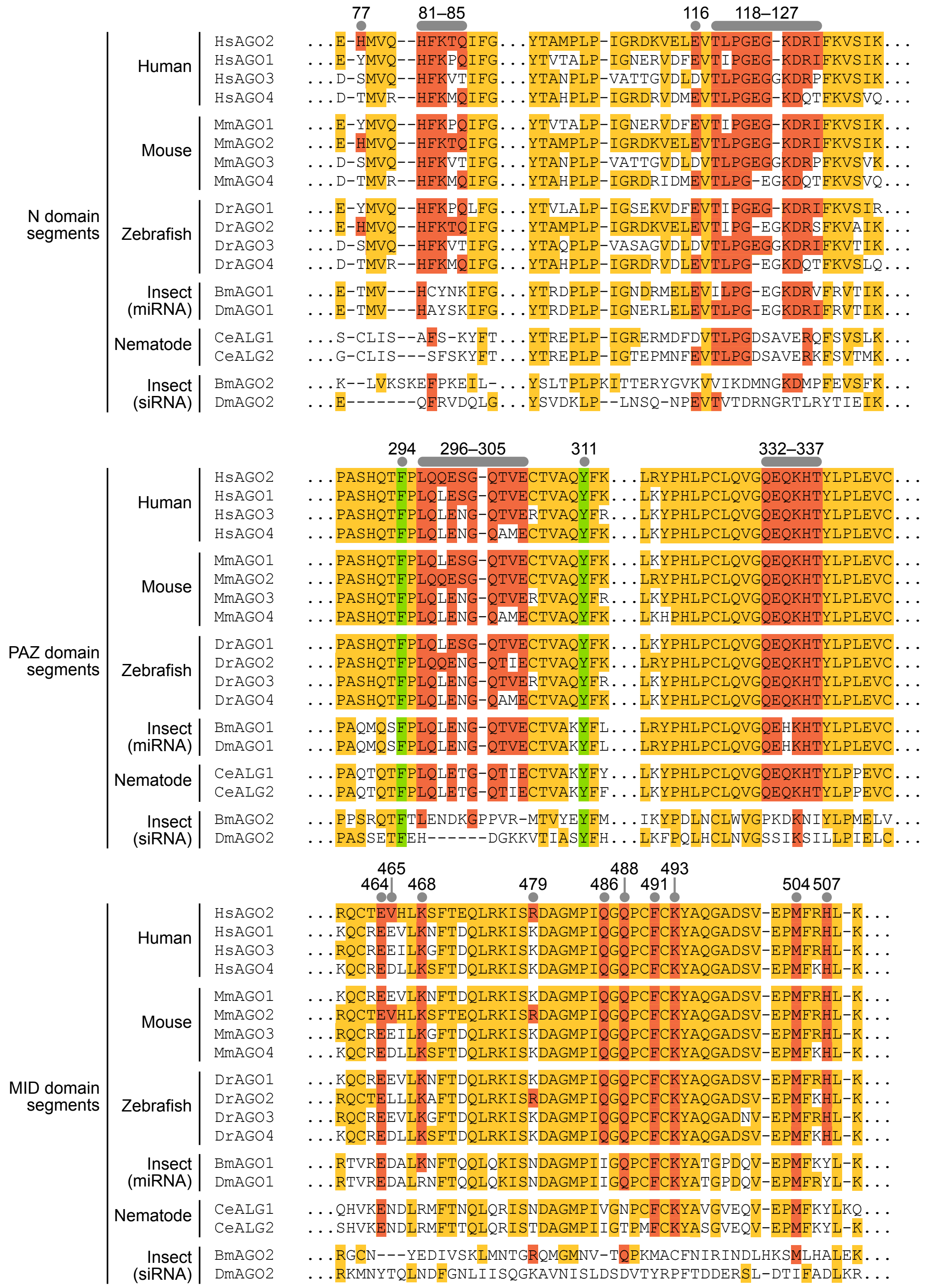

Supplementary Figure 11. Conservation of AGO protein sequence among homologs in diverse bilaterian species. Shown is a multiple-sequence alignment of regions that bind the ZSWIM8 protein, extracted from a sequence alignment of 111 AGO and PIWI homologs among metazoans, plants, fungi, and prokaryotes[76]. Residues identical to human AGO2 (HsAGO2) are highlighted in orange or red. Human AGO2 residues identified as ZSWIM8 contacts and assayed by substitutions are highlighted in red, as are homologous residues with conserved identity in other proteins. AGO residues identified as ZSWIM8 contacts are also marked with circles and with numbers indicating the position of the corresponding human AGO2 residue; F294 and Y311 were assayed to investigate the effect of a vacated PAZ pocket but not as ZSWIM8 contacts, and so are highlighted in green instead. For comparison, results for insect AGO2 are also included. Insect AGO2 arose from an evolutionary lineage distinct from that of insect AGO1 and other metazoan AGOs[90] (Supplementary Figure 10). It preferentially loads siRNAs instead of miRNAs and, based on studies of the fly protein[89], is not thought to be subject to TDMD.

bioRxiv preprint doi: https://doi.org/10.64898/2026.01.05.697729; this version posted January 5, 2026. The copyright holder for this preprint (which was not certified by peer review) is the author/funder, who has granted bioRxiv a license to display the preprint in perpetuity. It is made available under aCC-BY 4.0 International license.

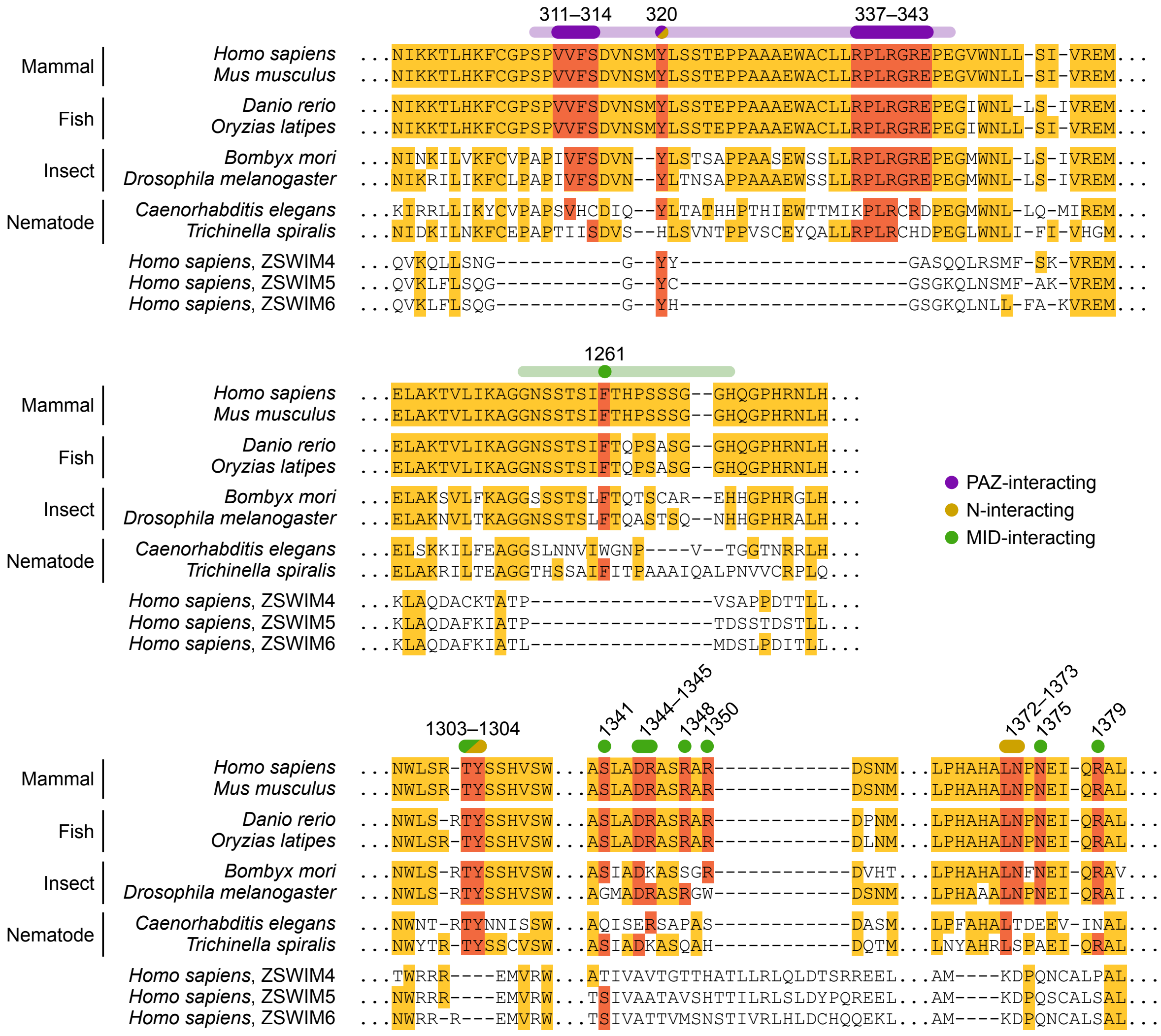

Supplementary Figure 12. Conservation of ZSWIM8 protein sequence among orthologs in diverse bilaterian species, Three human paralogs (ZSWIM4, ZSWIM5, and ZSWIM6) are also included for comparison. Shown is a multiple-sequence alignment of regions that bind the AGO protein, extracted from a sequence alignment of 1532 full-length ZSWIM4/5/6/8 metazoan homologs. Residues identical to human ZSWIM8 are highlighted in orange or red. Human ZSWIM8 residues that contact AGO are highlighted in red, as are homologous residues with conserved identity in other proteins. ZSWIM8 residues identified as AGO contacts are also marked with numbers indicating the position of the corresponding human ZSWIM8 residue, and with circles colored based on the domain of AGO with which they interact (key). ZSWIM4, 5, and 6, although related to ZSWIM8, are not thought to mediate TDMD. Indeed, ZSWIM8 residues that interact with AGO2 are mostly found in insertions unique to ZSWIM8 and not present in other members of the ZSWIM family.

bioRxiv preprint doi: https://doi.org/10.64898/2026.01.05.697729; this version posted January 5, 2026. The copyright holder for this preprint (which was not certified by peer review) is the author/funder, who has granted bioRxiv a license to display the preprint in perpetuity. It is made available under aCC-BY 4.0 International license.

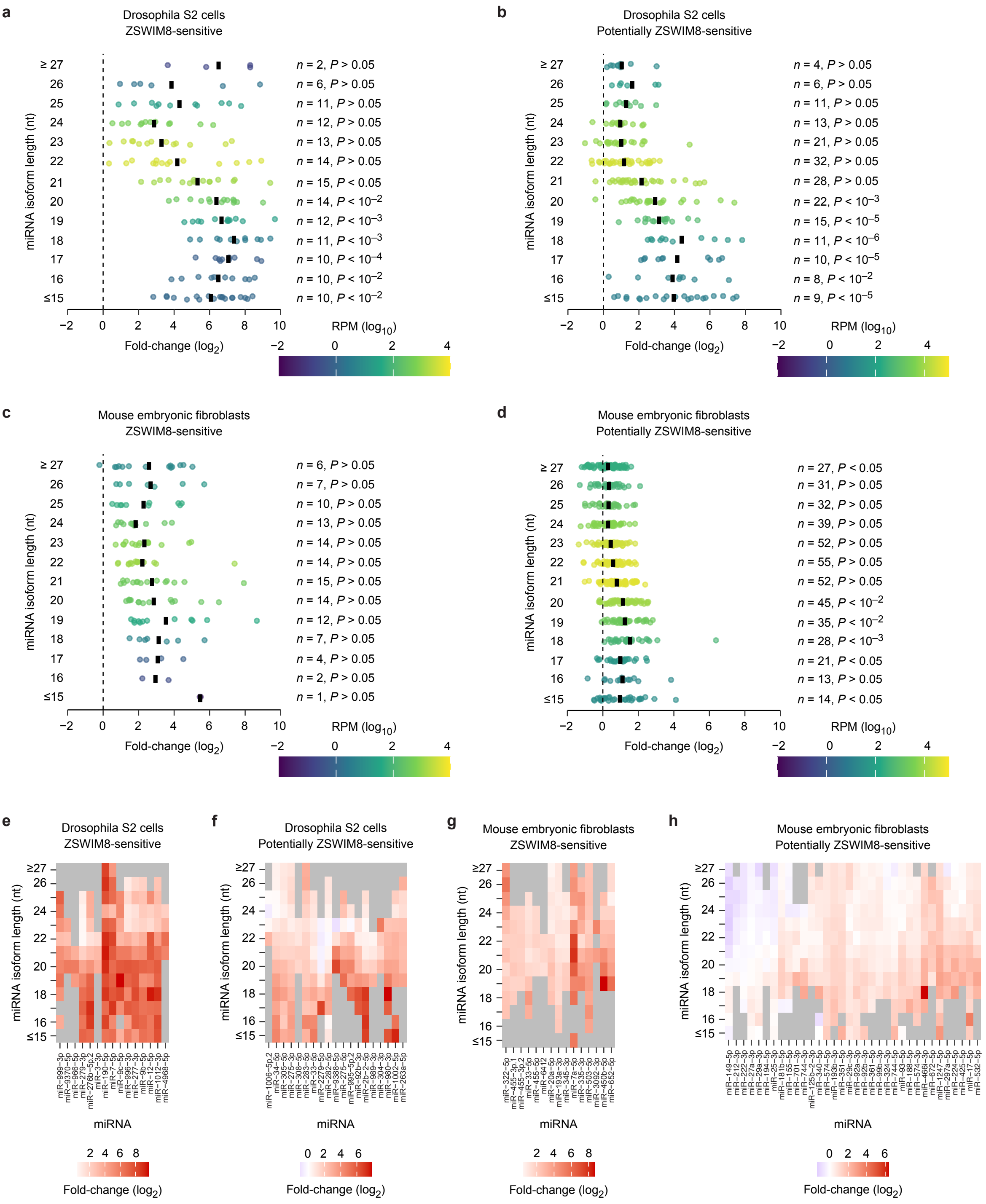

Supplementary Figure 13. Relationship between miRNA isoform length and ZSWIM8 sensitivity for ZSWIM8-sensitive miRNAs. **a**, Distribution of fold-changes ($\log_2$) of miRNA molecules of the indicated lengths in *Zswim8*-knockout versus control Drosophila S2 cells for ZSWIM8-sensitive miRNAs. This panel is as in Extended Data Figure 8a, except it considers miRNAs classified as accumulating significantly upon loss of ZSWIM8 by BBUM analysis (FDR-adjusted *P*-value < 0.05)[91]. Each point represents a unique miRNA isoform of a given length, and each vertical black line represents the mean of the distribution of miRNA isoforms of the indicated length. **b**, Distribution of fold-changes ($\log_2$) of miRNA molecules of the indicated lengths in *Zswim8*-knockout versus control Drosophila S2 cells for potentially ZSWIM8-sensitive miRNAs. This panel is as in **a**, except it considers miRNAs that fail to meet the more stringent cutoff for ZSWIM8 sensitivity in **a** and are instead classified as potentially ZSWIM8-sensitive based on meeting one of three criteria: 1) a $\log_2$ fold-change > 0 and $P_{adj} < 0.05$, 2) a $\log_2$ fold-change significantly larger than that of their passenger strands, or 3) previous annotation as ZSWIM8-sensitive in S2 cells or embryos[17,23]. **c**, Distribution of fold-changes ($\log_2$) of miRNA molecules of the indicated lengths in *Zswim8*-knockout versus control MEFs for ZSWIM8-sensitive miRNAs. This panel is as in **a**, except the analysis is of data from MEFs. **d**, Distribution of fold-changes ($\log_2$) of miRNA molecules of the indicated lengths in *Zswim8*-knockout versus control MEFs for potentially ZSWIM8-sensitive miRNAs. This panel is as in **b**, except the analysis is of data from MEFs, and miRNAs were classified according to previous annotations of ZSWIM8 sensitivity in mouse tissues[24]. **e**, Relationship between miRNA isoform length and ZSWIM8 sensitivity in Drosophila cells for ZSWIM8-sensitive miRNAs. This panel is as in Extended Data Figure 8c, except it considers miRNAs classified as ZSWIM8-sensitive, as in panel **a**. All ZSWIM8-sensitive miRNAs were included in the analysis, regardless of the number of isoforms passing the expression cutoff. **f**, Relationship between miRNA isoform length and ZSWIM8 sensitivity in Drosophila cells for potentially ZSWIM8-sensitive miRNAs. This panel is as in **e**, except it considers miRNAs classified as potentially ZSWIM8-sensitive, as in panel **b.** Results for all potentially ZSWIM8-sensitive miRNAs with at least four isoforms of different lengths are shown. **g**, Relationship between miRNA isoform length and ZSWIM8 sensitivity in MEFs for ZSWIM8-sensitive

bioRxiv preprint doi: https://doi.org/10.64898/2026.01.05.697729; this version posted January 5, 2026. The copyright holder for this preprint (which was not certified by peer review) is the author/funder, who has granted bioRxiv a license to display the preprint in perpetuity. It is made available under aCC-BY 4.0 International license.

miRNAs. This panel is as in **e**, except the analysis is of data from MEFs. All ZSWIM8-sensitive miRNAs were included in the analysis, regardless of the number of isoforms passing the expression cutoff. **h**, Relationship between miRNA isoform length and ZSWIM8 sensitivity in MEFs for potentially ZSWIM8-sensitive miRNAs. This panel is as in **f**, except the analysis is of data from MEFs. Results for all potentially ZSWIM8-sensitive miRNAs with at least six isoforms of different lengths are shown.

bioRxiv preprint doi: https://doi.org/10.64898/2026.01.05.697729; this version posted January 5, 2026. The copyright holder for this preprint (which was not certified by peer review) is the author/funder, who has granted bioRxiv a license to display the preprint in perpetuity. It is made available under aCC-BY 4.0 International license.

Supplementary References

86. Price, M. N., Dehal, P. S. & Arkin, A. P. FastTree 2 – Approximately Maximum-Likelihood Trees for Large Alignments. *PLOS ONE* **5**, e9490 (2010).
87. The UniProt Consortium. UniProt: the Universal Protein Knowledgebase in 2023. *Nucleic Acids Res.* **51**, D523–D531 (2023).
88. Edgar, R. C. MUSCLE: multiple sequence alignment with high accuracy and high throughput. *Nucleic Acids Res.* **32**, 1792–1797 (2004).
89. Kingston, E. R. & Bartel, D. P. Ago2 protects Drosophila siRNAs and microRNAs from target-directed degradation, even in the absence of 2′-O-methylation. *RNA* **27**, 710–724 (2021).
90. Swarts, D. C. *et al.* The evolutionary journey of Argonaute proteins. *Nat. Struct. Mol. Biol.* **21**, 743–753 (2014).
91. Wang, P. Y. & Bartel, D. P. A statistical approach for identifying primary substrates of ZSWIM8-mediated microRNA degradation in small-RNA sequencing data. *BMC Bioinformatics* **24**, 195 (2023).
